# Supramolecular Triblock Copolymers through the Formation of Hydrogen Bonds: Synthesis, Characterization, Association Effects in Solvents of Different Polarity

**DOI:** 10.3390/polym12020468

**Published:** 2020-02-18

**Authors:** Spyridoula-Lida Bitsi, Margarita Droulia, Marinos Pitsikalis

**Affiliations:** Industrial Chemistry Laboratory, Department of Chemistry, National and Kapodistrian University of Athens, Panepistimiopolis Zografou, 15771 Athens, Greece; lidabitsi@chem.uoa.gr (S.-L.B.); dmargarita_31@hotmail.com (M.D.)

**Keywords:** hydrogen bonds, supramolecular chemistry, self-assembly, anionic polymerization, solution properties, thermal properties

## Abstract

Anionic polymerization techniques were employed for the synthesis of linear polystyrene (PS) and block copolymer of PS and polyisoprene (PI) PS-*b*-PI bearing end hydroxyl groups. Following suitable organic chemistry transformation, the –OH end groups were converted to moieties able to form complementary hydrogen bonds including 2,6-diaminopurine, Dap, thymine, Thy, and the so-called Hamilton receptor, Ham. The formation of hydrogen bonds was examined between the polymers PS-Dap and PS-*b*-PI-Thy, along with the polymers PS-Ham and PS-*b*-PI-Thy. The conditions under which supramolecular triblock copolymers are formed and the possibility to form aggregates were examined both in solution and in the solid state using a variety of techniques such as ^1^H-NMR spectroscopy, size exclusion chromatography (SEC), dilute solution viscometry, dynamic light scattering (DLS), thermogravimetric analysis (TGA), differential thermogravimetry (DTG), and differential scanning calorimetry (DSC).

## 1. Introduction

Polymer chemistry has witnessed a real explosion over recent decades. A variety of living/controlled polymerization techniques have emerged over the years, which allows for the synthesis of macromolecules with controlled molecular weights, molecular weight distributions, stereochemistry, end-group functionalization, optical properties, etc. [1,2,3,4,5,6,7,8,9,10]. The employment of novel catalysts, linking, termination, and transfer agents, along with the use of recent advances in organic chemistry, e.g., Suzuki coupling, click chemistry, photochemical reactions etc., have led to the synthesis of complex well-defined macromolecular architectures [11,12,13]. An endless number of structures with numerous combinations of different polymer chains, topologies, and stereo-chemistries have emerged over the years, which shows that the macromolecular architecture can greatly affect the material properties both in solution and in the solid state [14,15,16,17,18,19].

All these studies have the common feature that the synthesized structures are based on stable covalent bonds. On the other hand, supramolecular chemistry is based on non-covalent interactions, such as π–π stacking, hydrogen bonds, ionic and electrostatic interactions, metal coordination chemistry, inclusion complexation, etc. [20]. The importance of this scientific field is shown by the fact that vital operations in nature are based on these types of supramolecular interactions. The stability of the double helix of DNA, molecular transportation, conformation, and, therefore, the biological activity of proteins and the evolution of the genetic information are a few examples of these types of operations that take place due to non-covalent interactions [21,22,23,24,25,26].

The combination of conventional covalent bond-based polymer chemistry with supramolecular chemistry opens new horizons in materials chemistry leading to very unique properties and various applications, such as reversible gels, shape-memory, and self-healing polymers [27,28,29,30,31]. Over the last two decades, particular interest has been given to the construction of complex macromolecular architectures based on non-covalent interactions and especially to the formation of hydrogen bonds between specially designed interacting groups covalently attached to polymer chains [32,33,34,35,36,37,38,39,40,41,42,43,44,45,46,47,48,49,50,51]. The synthetic accessibility, directionality, fidelity, and responsiveness to external stimuli (e.g., temperature, polarity of solvent, ionic strength, light, etc.) are the main advantages of these hydrogen bond-based supramolecular architectures. These stimuli-responsive materials possess properties that can be tuned by the external stimuli and properties such as processability, solution viscosity, and phase behaviour can be manipulated over a broad range of values [52,53].

In this work, we report the synthesis of supramolecular block copolymers PS_b_-*b*-PI-*b*-PS_a_, where PS is polystyrene and PI is polyisoprene, through the interaction of PS_a_ and PS_b_-*b*-PI linear chains bearing heterocomplementary hydrogen bond forming groups, namely 2,6-diaminopurine (Dap) and thymine (Thy) in the first case and Hamilton receptor (Ham) and Thy in the second case. These functions are very well-known interacting groups that have been frequently employed for the preparation of supramolecular polymers and the synthesis of supramolecular block copolymers [54,55,56,57,58]. A variety of experimental techniques such as Nuclear Magnetic Resonance spectroscopy (NMR), Size-Exclusion Chromatography (SEC), dilute solution viscometry, Dynamic Light Scattering (DLS), Differential Scanning Calorimetry (DSC), and Thermogravimetric Analysis (TGA) have been employed to verify the formation of the desired supramolecular structure and, furthermore, to study the association behavior, which is promoted by the polar hydrogen bonding interacting groups in non-polar solvents. The specific goals of this work are not only to establish efficient ways for the successful synthesis of the desired supramolecular block copolymers but also to study the hierarchy of structures that are formed in solution, which starts from the formation of the pseudo triblock copolymer through the formation of hydrogen bonds between the complementary groups. This leads to the formation of higher aggregates due to the association of the polar interacting groups in non-polar solvents.

## 2. Materials and Methods 

### 2.1. Materials

All manipulations were performed using high vacuum or Schleck techniques. The purification of solvents (benzene, dichloromethane, chloroform, methanol, and tetrahydrofuran (THF)) and monomers (ethylene oxide, isoprene, and styrene) was conducted according to the standards required by anionic polymerization, which are described elsewhere [59,60,61]. All materials were obtained from Merck (Darmstadt, Germany) and were used without further purification, unless mentioned otherwise. Triethylamine (TEA) was initially purified over calcium hydride, then over sodium metal, and was distilled directly into the Schleck tube of the specific reaction. 2,6-Diaminopyridine was recrystallized from boiling chloroform. The synthesis of the anionic initiator, *sec*-BuLi, was performed via the reaction of *sec*-BuCl with Li metal. Toluene and tetrahydrofuran (THF), used for the characterization, were refluxed from calcium hydride and metallic sodium, respectively, and distilled before use.

### 2.2. Polymer Synthesis

Conventional anionic polymerization high vacuum techniques were applied for the synthesis of PS-OH homopolymers and PS-*b*-PI-OH block-copolymers, according to the literature [61,62,63,64]. In order to introduce the hydroxyl group at the end of each polymeric chain, the polymerization was terminated initially, with a small amount of ethylene oxide, which was followed by the addition of methanol. Each polymer went through the process of freeze-drying before any further end group modification.

### 2.3. General Procedure for the Synthesis of Acrylated-Polymers

A Schleck tube was charged with the hydroxyl end-functionalized polymer, chloroform (10% w/v), and triethylamine, in a 1.8-fold excess over the acryloyl-chloride and was immersed into a 0 °C bath. Acryloyl-chloride, in a 10.0-fold excess over the end –OH groups, was subsequently added dropwise to this solution. The reaction was then stirred at room temperature for 48 h. The resulting yellowish solution was precipitated three times from CHCl_3_ in methanol. Lastly, the yellow crude product was dried under reduced pressure [65,66].

### 2.4. General Procedure for Michael Addition to Introduce the Heterocyclic Compounds to Acrylated Polymers (1,2)

A Schleck tube was charged with acrylated-polymer, heterocyclic compounds such as thymine (Thy) or 2,6-diaminopurine (Dap) in a 20.0-fold excess compared to the acrylated end group and a catalytic amount of potassium *tert*-butoxide in dry THF (8% w/v). The reaction was immersed in a 60°C oil bath and stirred for 24 h. The resulting yellowish solution was precipitated three times from CHCl_3_ in methanol. Lastly, the yellow crude product was dried under reduced pressure [65].

### 2.5. Synthesis of Bromo-Terminated PS (3)

In a Schlenck tube, a solution of triphenylphosphine (1.3 g, 5.0 mmol) in CH_2_Cl_2_ (13% w/v) was added dropwise to a mixture of PS-OH (10.0 g, 1.0 mmol), CBr_4_ (1.9 g, 5.8 mmol), and CH_2_Cl_2_ (5% w/v) in a 0 °C bath. The reaction was stirred at room temperature for 16 h. The solvent was completely removed under vacuum. The product was dissolved in toluene and filtered. The solvent was removed again, and the crude yellowish product was further purified by column chromatography (SiO_2_, diethyl ether/ethyl acetate 8:1, with a gradually increase of the proportion of diethyl ether) [67].

### 2.6. Synthesis of Bis[3,5-bis(methoxycarbonyl)phenoxy]-Terminated PS (4)

A Schleck tube was charged with bromo-terminated PS (9.0 g, 0.9 mmol), potassium carbonate (1.1 g, 8.1 mmol), 18-crown-6 (0.5 g, 1.8 mmol), and 5-hydroxyisophthalic acid dimethylester (1.9 g, 9 mmol) in dry THF (6% w/v). The reaction was immersed in a 70 °C oil bath and refluxed for 4 days. The solvent was then completely removed under vacuum, and the product was dissolved in CH_2_Cl_2_. The organic layer was washed three times with purified water, twice in brine, dried over calcium sulfate, and filtered. The produced brown solution was further purified by three precipitations, from CH_2_Cl_2_ solutions in methanol. Lastly, the yellow crude product was dried under reduced pressure [67].

### 2.7. Synthesis of Bis[3,5-bis(6-aminopyridin-2-yl-carbamoyl)phenoxy]-Terminated PS (5)

A three-neck round bottom flask was charged with a solution of 2,6-diaminopyridine (4.4 g, 40.0 mmol) in dry THF (750 mL), under argon atmosphere, at −78 °C (orange colour). A 2.5 M solution of *n*-butyllithium in hexane (14.4 mL, 36 mmol) was subsequently added dropwise and stirred for 30 min. A mixture of isophthalate-PS (4 g, 0.4 mmol), in dry THF was then added slowly at −78 °C, which was followed by stirring for 8 h. The mixture was then left at room temperature for an extra period of 13 h (vivid red colour). NaHCO_3(aq.)_ (40.0 mL, 1 M) was added to the solution to terminate the reaction (brown colour). The solvent was removed under vacuum, and the product was dissolved in CHCl_3_. The organic layer was washed three times with NaHCO_3(aq.)_, three times with brine, dried over calcium sulfate, and filtered. The resulting brown solution was further purified by three precipitations from CHCl_3_ solutions in methanol. Lastly, the yellow crude product was dried under reduced pressure [67].

### 2.8. Synthesis of Bis[3,5-bis[6-(butyrylamino)pyridin-2-yl-carbamoyl]-phenoxy]-Terminated PS (PS-Ham) (6)

A Schleck tube was charged with isophthalamide-PS (3.0 g, 0.3 mmol), chloroform (10% w/v), and trimethylamine (3.5 mL, 24.9 mmol) and was immersed in a 0 °C bath. Afterward, butyryl-chloride (1.2 g, 12.0 mmol) was added dropwise to the solution. The reaction was stirred at room temperature for 18 h. The solvent was completely removed under vacuum, and the product was dissolved in CHCl_3_. The organic layer was washed twice with NaHCO_3(aq.)_, once with brine, dried over calcium sulfate, and filtered. The resulting yellowish solution was precipitated three times from CHCl_3_ solutions in methanol. Lastly, the crude product was dried under reduced pressure [67].

### 2.9. Preparation of the Blends

The blends were prepared by mixing toluene solutions (at concentrations equal to 5% w/v) of PS-*b*-PI-Thymine and PS-2,6-Diaminopurine (Blend#1) or PS-*b*-PI-Thymine and PS-Hamilton (Blend#2), in a 1:1 ratio at room temperature. The solvent was, subsequently, gradually evaporated at room temperature and atmospheric pressure.

### 2.10. Characterization Techniques

Size exclusion chromatography (SEC) (Waters, Milford, USA) experiments were conducted at 40 °C using a modular instrument consisting of a Waters Model 510 pump, a Waters Model U6K sample injector, a Waters Model 401 differential refractometer, a Waters Model 486 UV spectrophotometer, and a set of 4 μ-Styragel columns with a continuous porosity range from 500 Å to10^5^ Å. The columns were housed in an oven thermostatted at 25 °C. CHCl_3_ was the carrier solvent at a flow rate of 1 mL/min. 

Dynamic light scattering (DLS) (Brookhaven, Holtsville, USA) measurements were performed with a Brookhaven Instruments Nanobrook Omni system operating at λ = 640 nm and with 40 mW power. Correlation functions were analyzed by the cumulant method and the Contin software. The correlation function was collected at 90°. A JDS Uniphase 22 mW He–Ne laser was used as the light source. The instrument was connected to a Polyscience model 9102 bath for temperature control, which allows measurements at a variable temperature. The time auto-correlation function *g_2_*(*q,t*) of the scattering intensity *I*(*q,t*) was calculated according to Equation (1) below [68].

(1) $$g_{2}\left(q,t\right)=\frac{\langle I\left(q,t\right)I\left(q,t+t_{0}\right)\rangle }{{\langle I\left(t\right)\rangle }^{2}}$$

where *t* is the time, *t*_0_ is the lag time, the <> operator denotes the average value, and *q* is the scattering wave vector.

(2) q=4πn0sin(θ2)λ

θis the observation angle or else the scattering angle between the incident light and the scattered light (angle of observation), *n*_0_ is the refractive index of the suspending medium, and λ is the wavelength of the incident light. Measurements were carried out five times for each concentration and were averaged. The solutions were filtered through 0.45-μm hydrophobic poly(tetrafluoroethylene) (PTFE) filters (Millex-LCR from Millipore) before measurements. The angle of the measurements was 90°. The experimental correlation functions were analyzed by the cumulants method and the CONTIN software [69]. Typically, the decay of the auto-correlation function is well captured by an exponential decay whose characteristic time depends on the diffusion coefficient of the scattering unit and yield a characteristic hydrodynamic size through the Stokes-Einstein-Sutherland equation. When different scattering populations exist in the sample, the analysis of the scattering signal reveals a distribution of relaxation times, which reflects different sizes.

Viscometric data were analyzed using the Huggins equation [70].

(3) $$\frac{\eta_{sp}}{c}=\left[\eta \right]+k_{H}{\left[\eta \right]}^{2}c+\ldots$$

and the Kraemer equation [17].

(4) $$\frac{ln\eta_{r}}{c}=\left[\eta \right]+k_{K}{\left[\eta \right]}^{2}c+\ldots$$

where *η_r_*, *η_sp_*, and [*η*] are the relative, specific, and intrinsic viscosities, respectively, whereas *k_H_* and *K_K_* are the Huggins and Kraemer constants, respectively. All measurements were carried out at 25 °C. Cannon-Ubbelohde dilution viscometers equipped with a Schott-Geräte AVS 410 automatic flow timer were used. 

The ^1^H-NMR spectra were recorded in d-chloroform at 305.0 K with a Bruker Avance III 600 NMR spectrometer (Bruker BioSpin, Rheinstetten, Germany).

Differential scanning calorimetry (DSC) experiments were performed with a 2910 Modulated DSC model from TA instruments (New Castle, USA). The samples were heated or cooled at a rate of 10 °C/min.

Thermogravimetric analysis (TGA) experiments were conducted with a Q50 model from TA instruments (New Castle, USA) under an inert atmosphere. The heating rate was adjusted to 10 °C/min.

## 3. Results

### 3.1. Polymer Synthesis and Characterization

**Figure 1:**
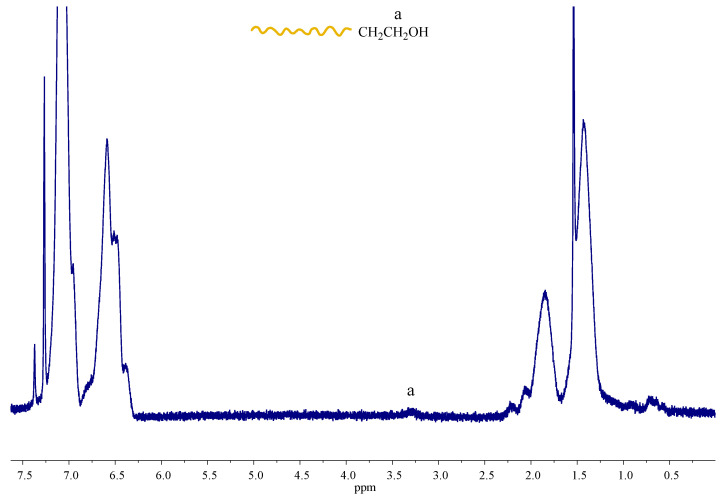
^1^H-NMR spectrum of sample PS-OH.

It is well known that anionic polymerization is a technique that allows the synthesis of polymers with narrow molecular weight distributions and predictable molecular weights [71]. By reacting the living polymers with ethylene oxide followed by quenching with methanol, the hydroxyl group can directly be attached at the end of each polymeric chain. With this approach, well-defined hydroxyl-terminated polymers with narrow molecular weight distributions were afforded, as confirmed by SEC and NMR measurements (Figure 1 and Figure S1 of the Supporting Information section). The molecular characteristics are given in Table 1. Both SEC measurements and end-group analysis by ^1^H-NMR resulted in similar molecular weights for the hydroxyl-terminated polymers. 

**Scheme 1:**
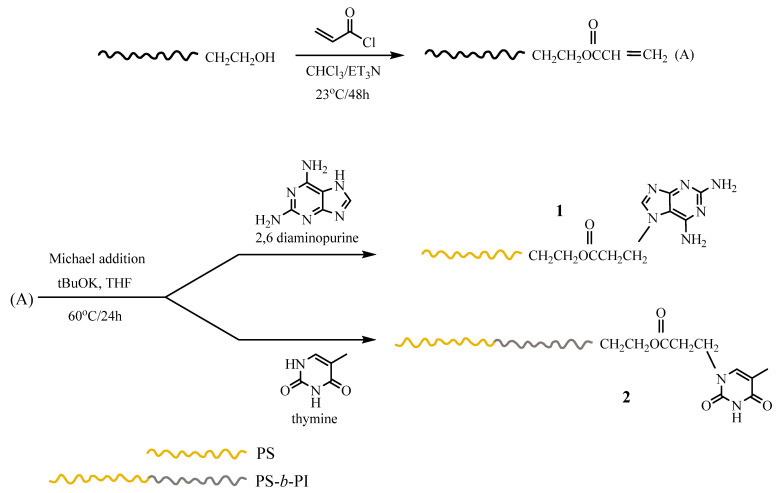
Synthetic route for PS-Dap (1) and PS-b-PI-Thy (2).

**Figure 2:**
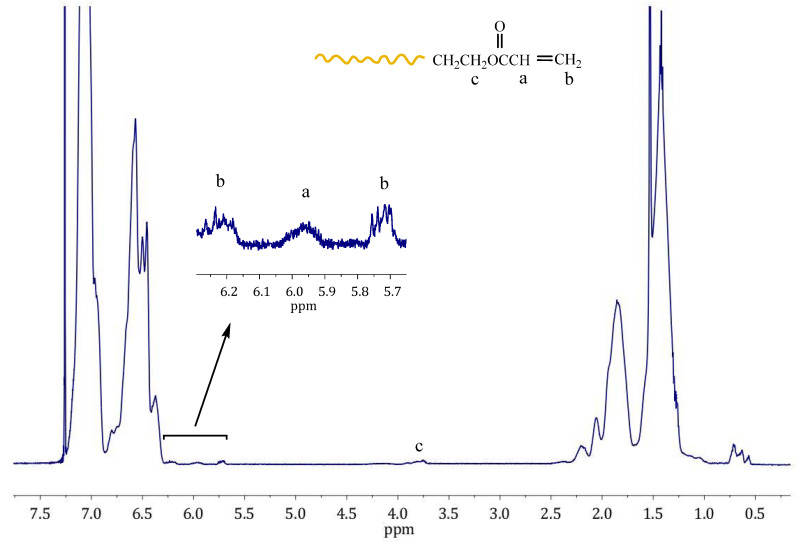
^1^H-NMR spectrum of sample PS-Acrylated.

**Figure 3:**
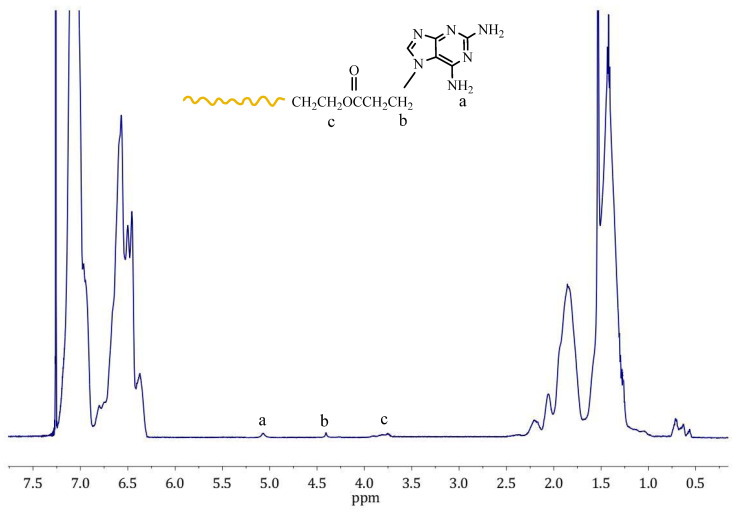
^1^H-NMR spectrum of sample PS-Dap.

**Figure 4:**
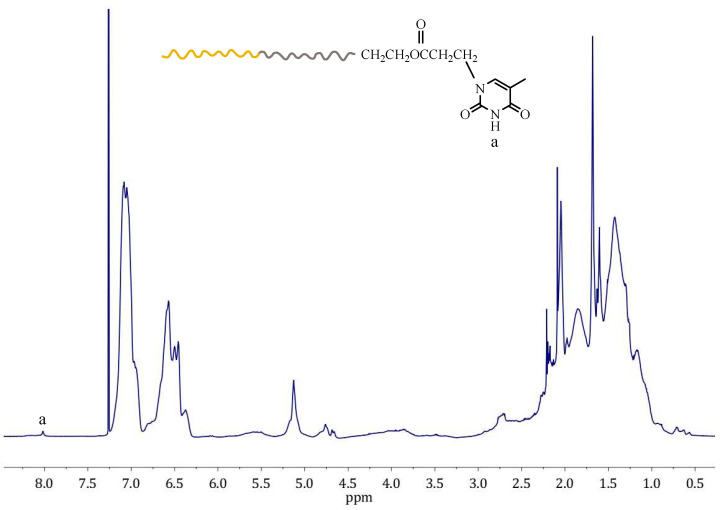
^1^H-NMR spectrum of sample PS-*b*-PI-Thy.

A two-step synthetic approach was carried out to introduce end-heterocyclic compounds, such as Thy or Dap, as shown in Scheme 1. The first step involves the introduction of an acrylated end group after the reaction of the end –OH group with acryloyl chloride in the presence of TEA, as reported in the literature [65,66]. ^1^H-NMR spectroscopy confirmed the efficiency of the synthetic procedure through the appearance of signals at 5.8–6.3 ppm, which were attributed to the three protons of the double bonds of the *H*_2_C=C*H*–COO– group, and, at 3.5–4.0 ppm, which were attributed to the two aliphatic protons –CH_2_C*H*_2_OOCCH=CH_2_ along with the disappearance of the peak at 3.3–3.5 ppm, which is assigned to the –CH_2_C*H*_2_OH of the precursor, as shown in Figure 2 and Figure S2. The second step was a Michael addition, from the acrylated end group in the presence of Thy or Dap and a catalytic amount of *t*-BuOK, to afford the heterocyclic terminated polymers. A catalytic amount of *t*-BuOK, which is a weak base, could expedite the Michael addition to yield the heterocyclic base [65]. The introduction of the end group was verified by ^1^H-NMR analysis. Dap-functionalized PS exhibited two distinct peaks corresponding to the Dap group at 5.2 ppm (a, –N*H_2_*) and 4.4 ppm (b, –C*H_2_*N–). The peaks corresponding to –N=C(–N*H_2_*)–N= and –N=C*H*–N– are overlapped with those of the polystyrene aromatic rings (Figure 3). In the case of the Thy end group of the PS-*b*-PI polymer, the peaks at 8.1 ppm (a, –CO–N*H*–CO–), 6.3 ppm [>N–C*H*=C(CH_3_)–] could be detected, along with the peaks at 3.8 ppm (–CH_2_C*H*_2_OOCCH_2_–) and 3.3 ppm (OOCCH_2_C*H_2_*-N) (Figure 4). Thy group can self-assemble, which results in the formation of dimers or larger aggregates [72,73,74,75]. A comparison of the *^1^*H-NMR spectra for PS-*b*-PI-acrylated and the corresponding PS-*b*-PI-Thy revealed a decrease in the polyisoprene composition due to the formation of aggregates in which the polyisoprene chains are located at the core of these aggregates and, thus, are less exposed to the magnetic field in the NMR instrument (Table 2). This will be further explained below.

**Scheme 2:**
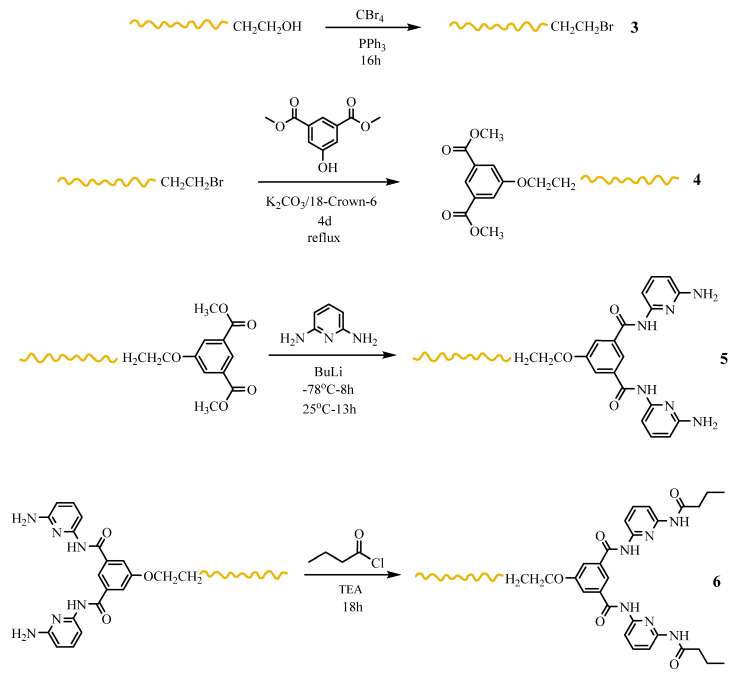
Synthetic route for PS-Ham (6).

**Scheme 3:**
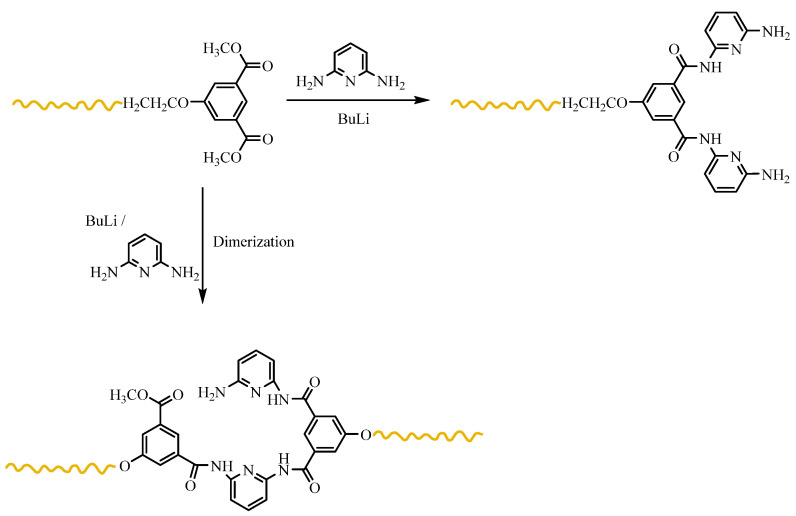
Dimerization of PS-Isophthalamide.

A four-step synthetic procedure (Scheme 2) was followed to introduce a chelate-type group at the PS’s end-group. Initially, PS-OH was transformed into PS-Br (**3**) upon reacting with CBr_4_ and triphenylphosphine. An SN_2-_type reaction took place to modify the PS-Br to the PS-isophthalate (**4**) using a combination of 18-crown-6 and K_2_CO_3_ in THF, which is followed by the addition of *n*-butyllithium and 2,6-diaminopyridine to introduce the isophthalamide (**5**) at the end of each polymeric chain. This was the most crucial step in this synthetic scheme due to the possibility of providing the dimer as a side product, as a result of an intermolecular reaction of two different polymer chains (Scheme 3), as described in the literature. This side reaction can be avoided by adding a slight excess of 2,6-diaminopyridine over the *n*-butyllithium in high dilution. SEC chromatography traces of the dimer were detected as a peak of lower elution volumes (Figure S3).

**Figure 5:**
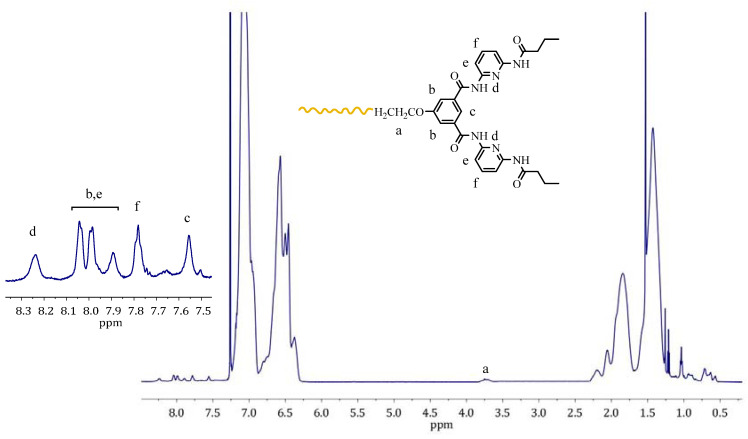
^1^H-NMR spectrum of sample PS-Ham.

The dimer was removed by fractionation, using toluene/methanol as a solvent/non-solvent system, and the procedure was monitored by SEC, as shown in Figure S4**.** Lastly, the desired end-functionalized PS-Ham (**6**) was produced by the reaction of isophthalamide-PS (**5**) with butyryl chloride and TEA in THF. A comparison of the ^1^H-NMR spectra, of the PS-OH and the corresponding products, confirms the successful synthesis of the desired PS-Ham (Figure 5). Specifically, six distinct peaks corresponding to the Ham group at 3.7 ppm (a, CH_2_C*H_2_*O–), 7.6 ppm (c, –CO=C–C*H*–C–CO–), 7.78 ppm (f, –C*H*=CH–C=N–), 7.8–8 ppm (b, –O–C=C*H*– and e, –CH=C*H*–C=N–), and 8.2 ppm (d, –CO–N*H*–) are clear in the NMR spectrum, M_n_ = 19,400 (calculated from the ^1^H-NMR integration ratio of aromatic protons at 6.3–7.5 ppm compared to –CO–N*H*– at 8.2 ppm).

### 3.2. ^1^H-NMR Analysis for the Blends

**Figure 6:**
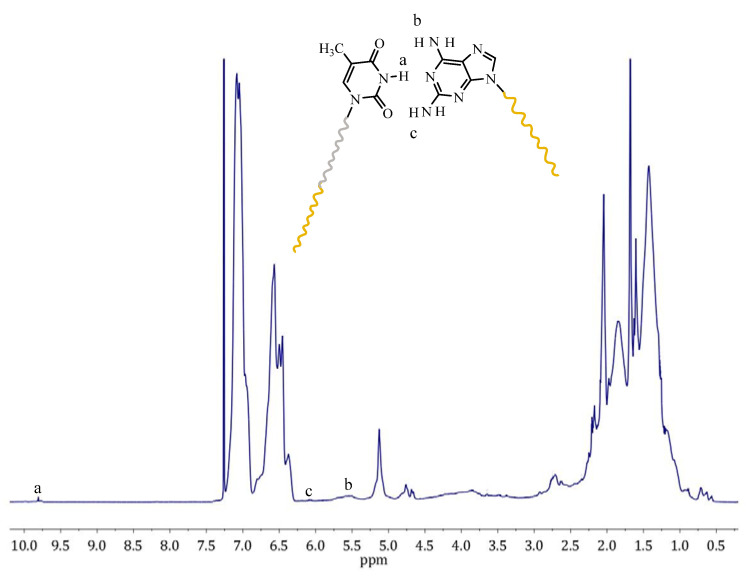
^1^H-NMR spectrum of sample Blend #1.

**Figure 7:**
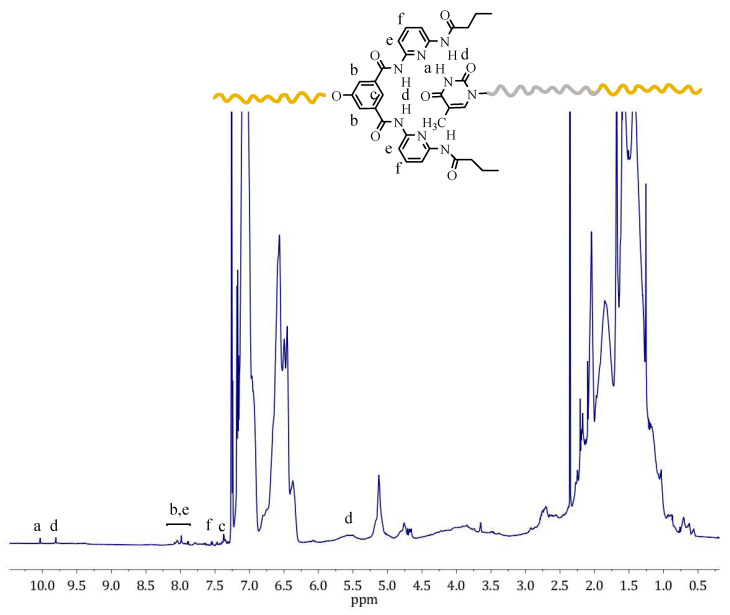
^1^H-NMR spectrum of sample Blend #2.

^1^H-NMR spectra for Blend #1 and Blend #2 are given in Figure 6 and Figure 7 in CDCl_3_ at 25 °C. It can be seen that the –NH peak of Thy at Blend #1 was shifted to a higher chemical shift from 8.1 to 9.8 ppm. The other hydrogen bonds are also detected, as shown in Figure 6, in agreement with literature data. Blend #2 presents a similar behavior since the –NH peak of Thy was shifted from 8.1 to 10.1 ppm. In addition, the signal from the –NH–C=O hydrogens of the Ham moiety were shifted from 8.25 to about 9.75 ppm (Figure 7). These shifts provide direct evidence of the hydrogen bonds formed in these two blends. As clear, both blends have similar ^1^H-NMR spectra, which is reasonable, since the precursors of the supramolecular structures (PS-OH and PS-*b*-PI-OH) were identical in both cases. The observed differences are related only to the presence of the functional end groups and the formation of the hydrogen bonds.

An additional observation that can be detected from the ^1^H-NMR spectra of the Blends is an apparent decrease of the polyisoprene composition in the PS-*b*-PI-Thy copolymer and the supramolecular triblock copolymers (Table 2). As mentioned previously, when the Thy group is attached to the PI block, aggregates begin to form, and, thus, the location of this block at the core of the forming aggregates renders the PI block less exposed to the magnetic field in the NMR instrument. The exact behaviour is exhibited in the blends. Due to the interactions between the two end groups of each blend, it can be speculated that a pseudo-triblock is formed in which the polyisoprene block is located in the middle, and, thereby, cannot be easily detected in the NMR spectrum. The reduced intensity of these peaks is direct evidence for the formation of the hydrogen bonds formed between the end groups of the polymers, which is further confirmed by the characterization data given below. 

### 3.3. Solution Properties

#### 3.3.1. Size-Exclusion Chromatography (SEC)

**Figure 8:**
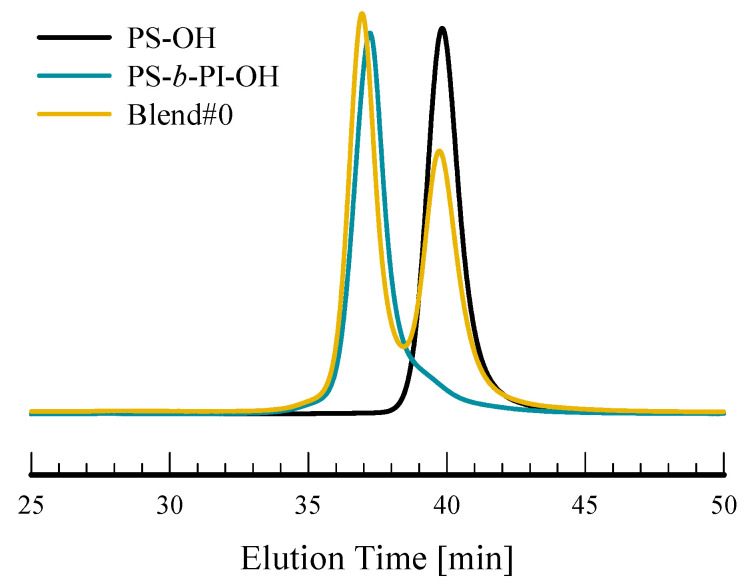
SEC traces of the samples PS-OH, PS-*b*-PI-OH, and Blend #0.

SEC is neither the crucial nor the major characterization technique for evaluating the formation of supramolecular structures through the presence of hydrogen bonds. These are delicate systems that can be influenced by changes in temperature, the shear forces applied in the separating columns, and the possible existence of aggregation phenomena. Under these circumstances, SEC cannot efficiently resolve the structures that are formed in solution and, therefore, it is not a suitable technique to provide quantitative information in supramolecular polymer chemistry. It is very rare in the literature to find SEC data supporting the synthesis of supramolecular polymers. NMR methodologies are the main tools to verify the existence of hydrogen bonds, which was shown in our case as well. However, in order to strengthen the conclusions regarding the presence of the hydrogen bond interactions, SEC experiments were performed for Blends #1 and #2. In addition, a different blend was created as well, which consisted of the hydroxyl functionalized precursors of the polymers that were used in the previous blends. Specifically, Blend #0 is a mixture of PS-OH and PS-*b*-PI-OH, prepared in the same way as Blends #1 and #2. To clarify the exact behaviour of these hydrogen bonded polymers, a variety of characterization methods were employed, starting with SEC in CHCl_3_. The SEC trace of Blend #0, shown in Figure 8, reveals the presence of two different narrow molecular weight distributed peaks. The one at the low elution time is attributed to the PS-OH and the other one, at higher elution time, and is attributed to PS-*b*-PI-OH, as a result of the lack of interaction between them. On the contrary, the SEC traces for Blend #1 and #2 are completely different from those of Blend #0, but are very similar to each other. 

**Figure 9:**
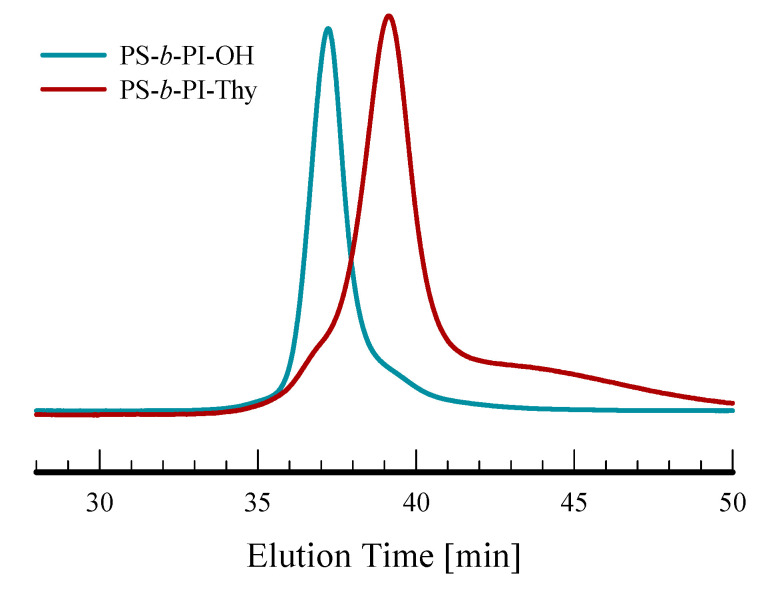
SEC traces of the samples PS-*b*-PI-OH and PS-*b*-PI-Thy.

SEC chromatography of the PS-*b*-PI-Thy (Figure 9) gives a trace at a higher elution volume when compared to its precursor. The presence of Thy leads to the formation of small and compact aggregates having smaller hydrodynamic volume, which elutes later than the –OH functionalized precursor. Except for the main peak, there are also small tails at lower elution volumes. Therefore, it can be assumed that this behavior is due to a dynamic equilibrium in solution between single chains, dimers, and a small population of higher aggregates, which are not stable enough against the external forces of the chromatography. The broad molecular weight distribution of the SEC trace reveals the presence of a mixture of products in solution that cannot be fully separated in the SEC columns. This result is only qualitative since aggregation phenomena cannot be quantitatively resolved by SEC.

**Figure 10:**
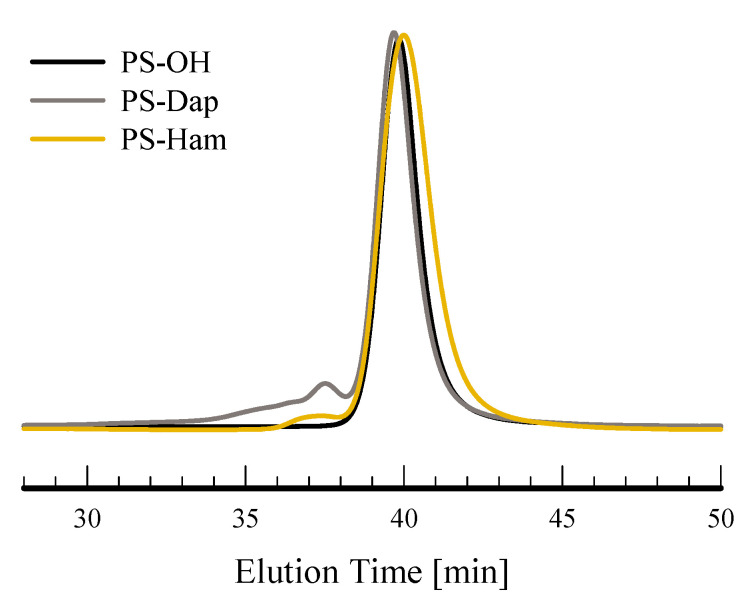
SEC traces of the samples PS-OH, PS-Dap, and PS-Ham.

PS-Ham and PS-Dap demonstrate similar behaviour, which is relatively different than that of PS-*b*-PI-Thy. The main peaks of PS-OH, PS-Dap, and PS-Ham, as shown in Figure 10, are approximately at the same elution volume. The difference exists when the end groups are attached to the polymers. The bulkiness of the end-groups and their chemical nature prevents the formation of large aggregates. In addition to the main peak, there are only minor shoulders at higher molecular weights, which can be explained as a small tendency for dimerization. This phenomenon is more evident in the PS-Dap, considering the tendency of the Dap end group to self-assemble more intensely than the Ham group, which forms more stable dimeric structures.

**Figure 11:**
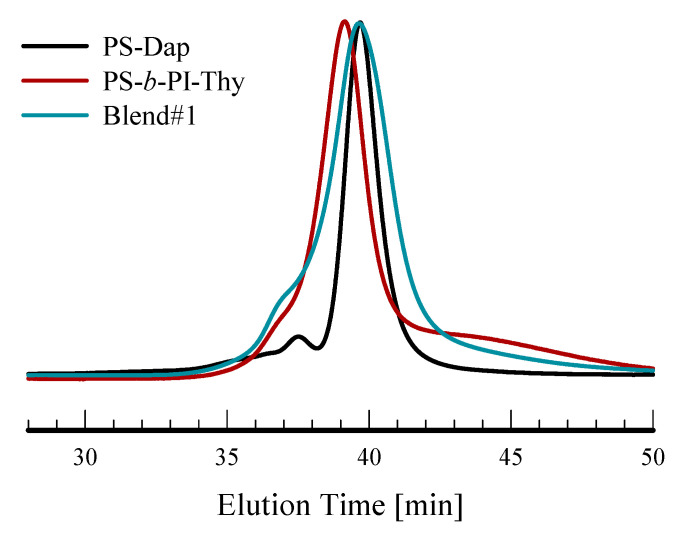
SEC traces of the samples PS-Dap, PS-*b*-PI-Thy, and Blend #1.

**Figure 12:**
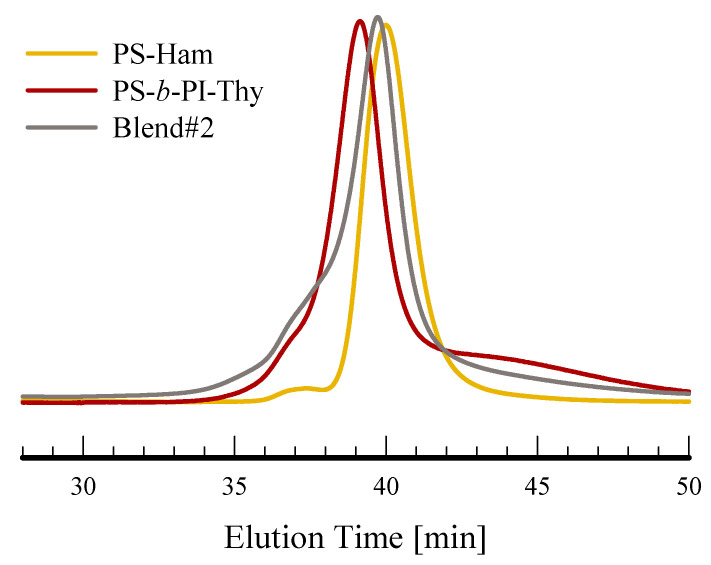
SEC traces of the samples PS-Ham, PS-*b*-PI-Thy, and Blend #2.

Considering the blends, the formation of the pseudo-tri-blocks is expected to manifest with the appearance of the SEC trace at lower elution volumes. However, this was not the case. An interesting observation regards the identical behavior of the two blends in SEC chromatography, which is a reasonable result due to the use of the same end hydroxyl polymers as the initial reagents for Blend #1 and #2. From Figure 11 and Figure 12, it is clear that there is one main peak in Blends #1 and #2, which is an indication that the solutions contain only one molecular species. This is clearly the supramolecular triblock copolymer in contrast to Blend #0, which is a solution of two different, non-interacting species. The main peak of the blends is located between the peaks of PS and PS-*b*-PI due to the change of the conformation of the constituting components upon the formation of the supramolecular structures. A more compact structure is present, which will be further conformed by intrinsic viscosity measurements. This indicates that compact structures of low viscosity prevail in solution. Traces in lower elution times are a direct indication of the coexistence of higher aggregates. However, these aggregations are not stable enough, and most of them are disrupted under the employment of the shear forces in the separation columns. This is direct evidence for the formation of the supramolecular desired structures, which was also revealed by NMR spectroscopy.

#### 3.3.2. Dilute Solution Viscometry

**Figure 13:**
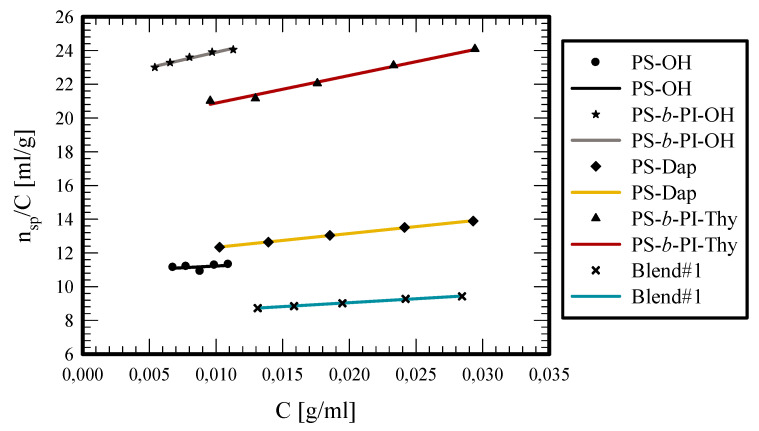
Huggins plots for samples PS-OH, PS-*b*-PI-OH, PS-Dap, PS-*b*-PI-Thy, and Blend #1.

**Figure 14:**
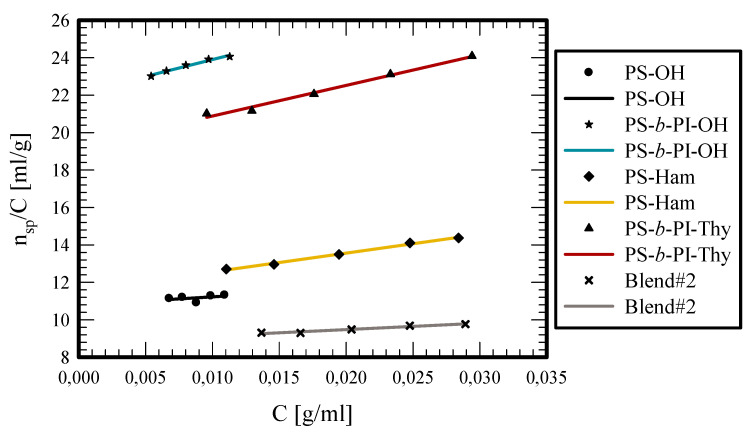
Huggins plots for samples PS-OH, PS-*b*-PI-OH, PS-Ham, PS-*b*-PI-Thy, and Blend #2.

Viscometry measurements were also performed in toluene, which is a non-polar solvent. Thus, this allows for the formation of hydrogen bonds. The results are given in Table 3, and representative plots are displayed in Figure 13, Figure 14 and Figure S5.

As observed in SEC measurements, the blends had a different behavior in their hydrodynamic volume than expected. A similar behaviour was observed in viscometry. The intrinsic viscosity for the samples PS-OH and PS-*b*-PI-OH are 10.87 and 22.09 mL/g, respectively. The low Huggins constants of these polymers reveal the absence of any kind of interactions between the –OH groups, which is very reasonable, since they are not polar enough to promote association. When the functional groups (Dap, Ham, and Thy) are located at the polymer chain ends, the intrinsic viscosities are approximately the same as their precursors, considering that the end groups are very small relative to the molecular weight of the polymers. Therefore, their contribution is not pronounced enough to promote any change. However, the Huggins constants were higher than the precursors indicating weaker interactions of the polymers with the solvent. PS*-b*-PI-Thy shows the smaller change in the *K_H_* value since it has the higher molecular weight and, therefore, the effect of the end-group is less pronounced than the other samples. On the other hand, PS-Dap and PS-Ham exhibit much higher Hugging constants. The low molecular weight of these samples reveals that the contribution of the end-groups is much more pronounced, which leads to increased interactions between the end-polar groups. Their interactions are not strong enough to endure the shear forces applied in the capillary tube of the viscometer and, thus, lead to increased intrinsic viscosities.

The experimental results for Blend #0 reveal that there are no interactions between the two polymers. This is manifested by the intrinsic viscosity, which lies between the values of PS-OH and PS-*b*-PI-OH and the Hugging constant that is almost the same value. A different situation was observed for Blend #1 and #2. Nonetheless, both samples had exactly the same behaviour. For both samples, the intrinsic viscosities’ values are much lower than their precursors. The results are in agreement with the NMR and SEC data confirming that the supramolecular structures behave more or less as hard spheres having smaller hydrodynamic volumes and lower intrinsic viscosities than their linear counterparts. Lastly, it is clear that the Huggins constants for Blends #1 and #2 are very high. This behavior can be attributed to the presence of the hydrogen bonds that form between the end functional groups of each blend. As a result, the structures behave as unimolecular micelles with more compact structures compared to the corresponding linear ones.

#### 3.3.3. Dynamic Light Scattering (DLS)

Dynamic light scattering (DLS) studies were conducted in THF, which is a moderately polar solvent. This prevents the formation of hydrogen bonds, and, in toluene, a non-polar solvent, which allows the formation of hydrogen bonds. The measurements were conducted at 25 °C using a 632.8-nm laser source at 90°. Different concentrations were employed for each sample. In all cases, the dependence of the apparent values of R_h_ did not vary appreciably with concentration. The time-correlation functions were analyzed by the CONTIN program. 

**Figure 15:**
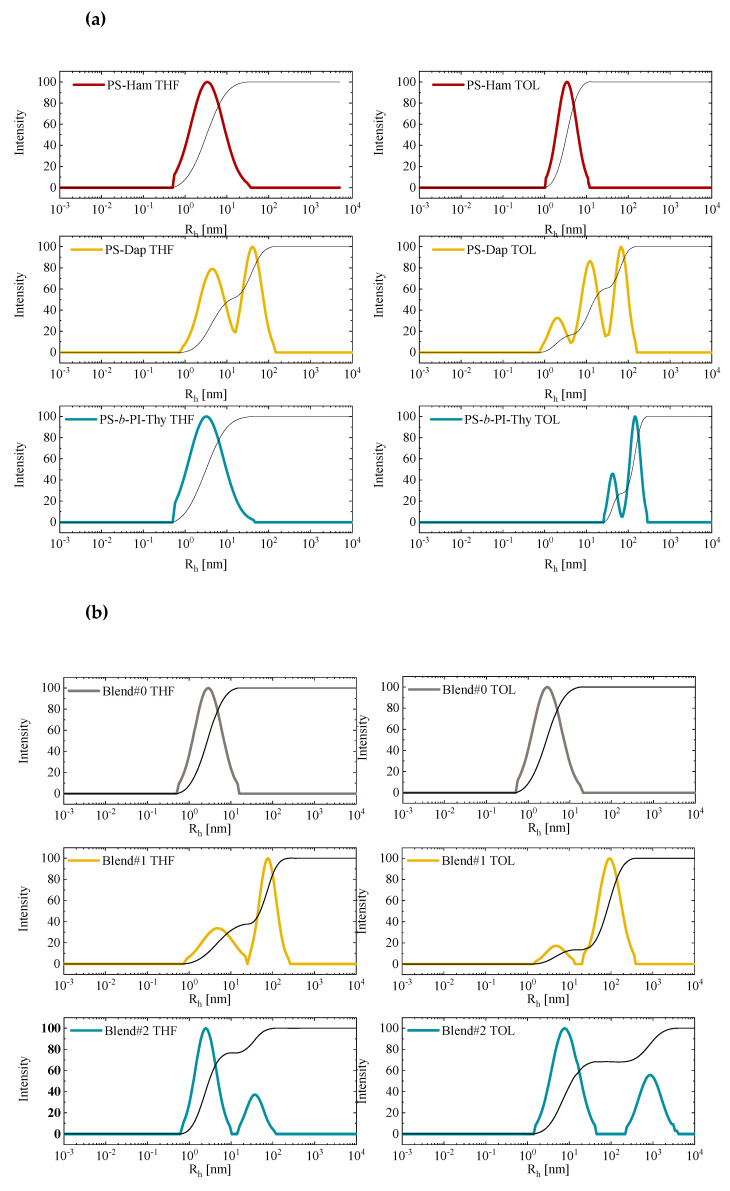
CONTIN analysis of PS-Ham, PS-Dap, PS-*b*-PI-Thy, (**a**) and of Blend #0, Blend #1, and Blend #2 (**b**) in THF and toluene, at 25 °C.

The analysis for Blend #0 revealed the absence of any kind of association in either THF or in Toluene. This result is in excellent agreement with the previously reported data from SEC and dilute solution viscometry measurements, which indicate, once more, that the end –OH groups are not polar enough and do not interact strongly to promote association. However, the situation is different when end-polar groups are covalently attached to non-polar polymer chains. These products may behave as amphiphilic systems, which leads to the formation of aggregates in non-polar solvents. The final solution behaviour will depend on the polarity of the end group, the molecular weight of the non-polar macromolecular chain, and the polarity of the solvent. The results from the DLS measurements are provided in Table 4, whereas characteristic CONTIN plots are provided in Figure 15. 

The PS-Ham sample, both in THF and in toluene, shows one population of a low R_h_ value. It seems that the bulky nature of the end group prevents the formation of higher aggregates in both solvents. However, the higher R_h_ value measured in toluene (4.77 nm against 2.07 nm in THF) and the lower polydispersity value from the CONTIN analysis in toluene indicate the formation of more compact dimeric structures in toluene.

The PS-Dap sample, on the other hand, showed pronounced differences compared to the PS-Ham sample. Strong association effects were obtained both in THF and in toluene. The smaller size and the more polar nature of the Dap end group, due to the presence of primary –NH_2_ groups, compared to the Ham end group promote much higher degrees of association in solution. Actually, the situation is more complex in toluene since three different populations are observed in this solvent. The *R_h_* values of the various populations are higher in toluene, and, most importantly, the contribution of the large aggregates is much more pronounced in toluene than in THF, which indicates that the relatively polar nature of THF prevents the formation of higher aggregates. 

Lastly, the PS-*b*-PI-Thy sample showed a distinct difference in the two solvents of different polarity. A single population of low *R*_h_ value is observed in THF. The relatively polar nature of the solvent along with the high molecular weight of the di-block copolymer prevent the formation of aggregates. On the contrary, from CONTIN analysis, the non-polar toluene promotes strong association with the appearance of two distinct populations with both having large R_h_ values.

The Blends #1 and #2 were also studied in THF and in toluene. In toluene, supramolecular triblock copolymers exist. The internal hydrogen bonded groups may be able to promote association due to their polar nature, which was already indicated from the DLS studies of the individual polymeric components bearing the end-polar groups Dap and Thy. In THF, the hydrogen bonds are not stable and, therefore, the supramolecular structures are broken down into their constituent components. However, even in THF, association is also expected for the supramolecular structures that still remain in solution and from the end-functionalized polymers that are formed after the disruption of the hydrogen bonds, as discussed earlier. Taking into account these statements, it is possible to understand the DLS measurements and, therefore, the solution behaviour of the blends. 

CONTIN analysis of the results coming from Blend #1 in toluene indicates the presence of two distinct populations. The first, with a low R_h_ value, is attributed to the free supramolecular structures, whereas the second population, with a high R_h_ value, is attributed to associations of the supramolecular structures. The aggregates prevail over the free supramolecular tri-blocks, as shown by the relative area of the two peaks from CONTIN analysis. In THF, two populations are also obtained from CONTIN analysis. The first population is attributed to possible remaining supramolecular tri-blocks and to the PS-*b*-PI-Thy components since both have R_h_ values very close to the R_h_ value of this peak. The second peak, of high R_h_ value, is attributed to aggregates from remaining supramolecular structures and to mixed aggregates from PS-Dap and PS-*b*-PI-Thy linear chains. It should be noted that, in THF, the second population is less pronounced compared to that in toluene and has a lower R_h_ value due to the less extended association phenomena in THF.

The aggregation effects are more clear in the case of Blend #2 in toluene. Judging from the relative size of the two populations, equilibrium between aggregates and clusters can be observed. The micellar structures prevail over the clusters, which can be concluded from the areas of the two populations by CONTIN analysis. The stronger aggregation phenomena in Blend #2 compared to Blend #1 can be attributed to the stronger interactions between the interacting groups in the first case (Ham against Thy groups) and their larger size, which facilitates the accommodation of additional supramolecular tri-blocks to micellar structures. The association phenomena in THF are much less intense. CONTIN analysis reveals that the major population is of very low R_h_ value, which corresponds to surviving supramolecular copolymers and their components, whereas the second minor population indicates the presence of micellar structures coming from the remaining intact supramolecular tri-block copolymers.

### 3.4. Thermal Properties

#### 3.4.1. Thermogravimetric Analysis (TGA)

As presented in Table 5**,** all the precursors of the blends (PS-OH, PS-*b*-PI-OH, PS-Dap, PS-Ham, and PS-*b*-PI-Thy) have the characteristic decomposition temperatures of the blocks of PS and PI without any substantial difference. Blend #0 also exhibits the same behaviour. It is associated with two different decomposition temperatures—low temperatures of the first step, attributed to the thermal decomposition of the PI block, and higher temperatures of the second step, attributed to the corresponding PS block. The decomposition peak of the PI component appears as a shoulder close to the main decomposition step of the PS component. This is reasonable since PI is the minor component in the blend.

**Figure 16:**
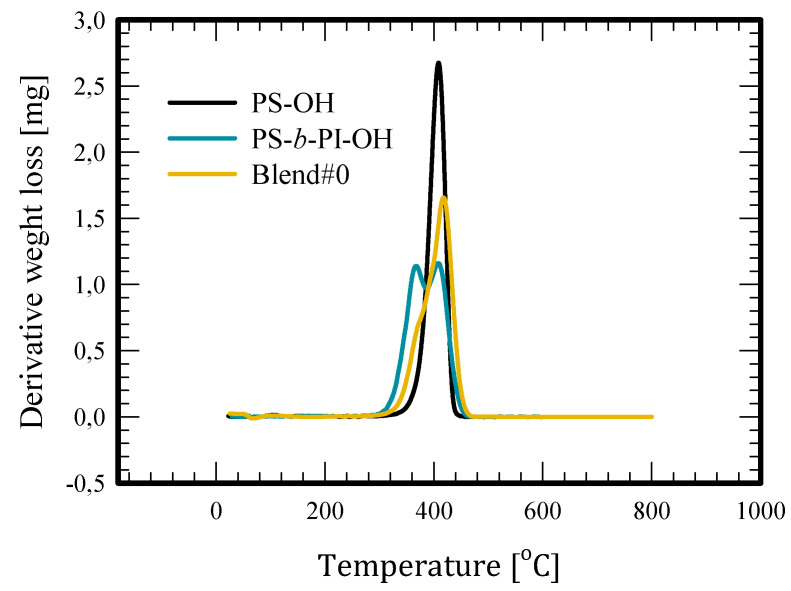
Differential Thermogravimetry (DTG) plots of the samples PS-OH, PS-*b*-PI-OH, and Blend#0.

On the contrary, Blends #1 and #2 reveal different decomposition steps (Figure 16, Figure S6, and Figure S7). The first step is at approximately 180 °C and indicates the disruption of the hydrogen bonds and the thermal decomposition of the end groups. For the PI block, there is no distinct decomposition temperature. Differential thermogravimetry reveals only a shoulder at lower temperatures, which is an indication of the thermal decomposition of the PI block. For the PS block, which is the major component, the decomposition temperature is found as expected from the previously mentioned control experiments. Lastly, there is also a third decomposition step at even higher temperatures. In Blend #1, it is seen as a small shoulder but is clearer in the case of Blend #2. This step can be attributed to the thermal decomposition of the aggregates that are formed in the solid state. The presence of these aggregates, especially in the case of Blend #2, was manifested by DLS measurements and is expected to be even more pronounced in the solid state.

#### 3.4.2. Differential Scanning Calorimeter (DSC)

Further evidence regarding the presence of hydrogen bonds in the solid state was provided by differential scanning calorimetry (DSC), and the measurements are summarized in Table 6.

The *T*_g_ values for the samples PS-OH, PS-Dap, and PS-Ham are approximately the same, as expected for PS 10 K, and no indication of any appreciable association is clear. The PS-*b*-PI-OH copolymer exhibited three transitions due to partial mixing of the PS and PI phases, and the appearance of a third phase, which is attributed to the extended interphase between the individual PS and PI phases. The rather low molecular weights of the PS and PI blocks (located at the weak segregation limit) are responsible for this behaviour. On the other hand, the PS*-b*-PI-Thy copolymer showed only two *T*_g_ values. Partial mixing of the PS and PI phases still exist in this case, but the interphase region is not pronounced enough to provide a third *T*_g_ value. The formation of the extended interphase is prohibited in this case due to the formation of aggregates through the interaction of the end Thy groups. Blend #0 also provides two *T*_g_ values. One is attributed to the PI component that has almost the same value as in the case of the PS-*b*-PI-Thy copolymer. However, the *T*_g_ attributed to the PS component is higher due to the presence of pure linear PS in the blend and to the increased total composition in PS. The blends #1 and #2 show an identical behaviour. In these blends, supramolecular tri-block copolymers exist together with their aggregates. Therefore, the interaction of the PS and PI phases is more effective, which leads to a better mixing and, thus, to *T*_g_ values that are closer to each other compared to that of Blend #0.

## 4. Conclusions

High-vacuum anionic polymerization techniques were used to prepare linear polystyrene, PS, and block copolymer of PS and polyisoprene, PI, PS-*b*-PI bearing end hydroxyl groups. These –OH precursors were further transformed to groups that are able to form complementary hydrogen bonds, namely 2,6-diaminopurine, Dap, thymine, Thy, and the Hamilton receptor, Ham. The formation of hydrogen bonds was examined between the polymers PS-Dap and PS-*b*-PI-Thy, along with the polymers PS-Ham and PS-*b*-PI-Thy. ^1^H-NMR spectroscopy in CDCl_3_ revealed the formation of supramolecular tri-block copolymers in both cases. This conclusion was also verified by size exclusion chromatography, SEC, and dilute solution viscometry. Dynamic light scattering, DLS measurements, and the CONTIN analysis showed that the polar end-groups of the individual polymer chains and the supramolecular tri-blocks are able to promote the formation of aggregates in solution. This behaviour is more pronounced in the non-polar solvent toluene versus the relatively polar solvent THF. The thermal properties of these hydrogen-bonded interacting samples was further examined by thermogravimetric analysis, TGA, differential thermogravimetry, DTG, and differential scanning calorimetry (DSC).

## Figures and Tables

**Table 1:** Molecular characteristics of the end-functionalized polymers.

SAMPLE	M_n_^a^	M_n_^b^	Ð^a^	%mol PS^b^	%mol PI^b^ (ratio of 1,4 over 3,4 units)
PS-OH	11,600	10,900	1.07	100	-
PS-*b*-PI-OH	21,500	20,900	1.08	42.3	57.7 (92.3/7.7)

^a^ by SEC in CHCl_3_ at 25 °C, ^b^ by ^1^H-NMR in CHCl_3_ at 25 °C.

**Table 2:** PS and PI composition in the modified diblocks and blends.

SAMPLE	%mol PS^a^	%mol PI^a^	%mol 1,4 PI^a^	%mol 3,4 PI^a^
PS-*b*-PI-OH	42.3	57.7	92.3	7.7
PS-*b*-PI-Acrylated	45.4	54.6	92.8	7.2
PS-*b*-PI-Thy	76.0	24.0	88.6	11.4
Blend #1	78.0	22.0	81.8	18.2
Blend #2	84.4	15.6	81.3	18.7

^a^ by ^1^H-NMR in CHCl_3_ at 25 °C.

**Table 3:** Viscometry results of the end-functionalized polymers and the blends in Toluene at 25 °C.

SAMPLE	[*η*]_H_ [mL/g]	*k_H_*
PS-OH	10.87	0.36
PS-Dap	11.51	0.62
PS-Ham	11.56	0.75
PS-*b*-PI-OH	22.09	0.37
PS-*b*-PI-Thy	19.25	0.44
Blend #0	18.14	0.40
Blend #1	8.11	0.71
Blend #2	8.80	0.72

**Table 4:** DLS results for various samples in THF and toluene (values in parentheses indicate the percentage of each population from CONTIN analysis).

Sample	Solvent	R_h01_ [nm]	R_h02_ [nm]	R_h03_ [nm]
PS-Dap	THF	1.48 (53%)	15.71 (47%)	-
	Toluene	4.81 (18%)	8.52 (44%)	14.68 (38%)
PS-Ham	THF	2.07 (100%)	-	-
	Toluene	4.77 (100%)	-	-
PS-*b*-PI-Thy	THF	3.64 (100%)	-	-
	Toluene	31.44 (27%)	97.51 (73%)	-
Blend #0	THF	3.59 (100%)	-	-
	Toluene	3.90 (100%)	-	-
Blend #1	THF	4.70 (39%)	64.63 (61%)	-
	Toluene	4.71 (15%)	79.55 (85%)	
Blend #2	THF	4.95 (78%)	14.95 (22%)	-
	Toluene	54.41 (69%)	445.19 (31%)	-

**Table 5:** TGA results of the end-functionalized polymers and the blends.

Sample	Maximum Temperature of Thermal Decomposition [°C]			Temperature Region of Thermal Decomposition [°C]
PS-OH	410			319–456
PS-2,6-Dap	414			346–454
PS-Ham	410			317–453
PS-*b*-PI-OH	374		410	280–473
PS-*b*-PI-Thy	364		411	252–464
Blend #0	414			290–464
Blend #1	189	418	454	125–512
Blend #2	177	419	512	128–570

**Table 6:** DSC results for the end-functionalized polymers and blends.

Sample	*T*_g_ [°C]			
PS-OH	96			
PS-Dap	97			
PS-Ham	100			
PS-*b*-PI-OH	−57	38		75
PS-*b*-PI-Thy	−60		57	
Blend #0	−63		88	
Blend #1	−50		75	
Blend #2	−51		71	

## Supplementary Materials

The following are available online at https://www.mdpi.com/2073-4360/12/2/468/s1: Figure S1: ^1^H-NMR spectrum of sample PS-*b*-PI-OH. Figure S2: ^1^H-NMR spectrum of sample PS-*b*-PI-Acrylated. Figure S3: Monitoring the synthesis of PS-Ham by SEC chromatography, in CHCl_3_ at 25 °C. Figure S4: PS-Isophthalamide before and after fractionation, by SEC chromatography, in CHCl_3_ at 25 °C. Figure S5: Huggins plots for samples Blend #0, Blend #1, and Blend #2. Figure S6: DTG plots of the samples PS-Dap, PS-*b*-PI-Thy, and Blend #1. Figure S7: DTG plots of the samples PS-Ham, PS-*b*-PI-Thy, and Blend #2.
