# Correction: Bitsi et al. Supramolecular Triblock Copolymers through the Formation of Hydrogen Bonds: Synthesis, Characterization, Association Effects in Solvents of Different Polarity. *Polymers* 2020, *12*, 468

**DOI:** 10.3390/polym14153219

**Published:** 2022-08-08

**Authors:** Spyridoula-Lida Bitsi, Margarita Droulia, Marinos Pitsikalis

**Affiliations:** Industrial Chemistry Laboratory, Department of Chemistry, National and Kapodistrian University of Athens, Panepistimiopolis Zografou, 15771 Athens, Greece

The authors wish to make the following corrections to the published paper [1]. They should be as follows:

In the original publication, the Acknowledgments section “Acknowledgments: The present work was funded through the Operational Program “Human Resources Development, Education and Lifelong Learning,” project title “Macromolecular architecture via hydrogen bonds. Synthesis, Characterization, Properties.” (project code: 5006393).” should be changed to “Acknowledgments: This research is co-financed by Greece and the European Union (European Social Fund—ESF) through the Operational Programme «Human Resources Development, Education and Lifelong Learning 2014–2020» in the context of the project “Macromolecular architecture via hydrogen bonds. Synthesis, Characterization, Properties.” (MIS 5006393).”

The authors apologize for any inconvenience caused and state that the scientific conclusions are unaffected. This correction was approved by the Academic Editor. The original publication has also been updated.
